# Predelivery Haemostatic Biomarkers in Women with Non-Severe Postpartum Haemorrhage

**DOI:** 10.3390/jcm13144231

**Published:** 2024-07-19

**Authors:** Claire de Moreuil, Brigitte Pan-Petesch, Dino Mehic, Daniel Kraemmer, Theresa Schramm, Casilda Albert, Christophe Trémouilhac, Sandy Lucier, Hubert Galinat, Liana Le Roux, Johanna Gebhart, Francis Couturaud, Alisa S. Wolberg, Cihan Ay, Ingrid Pabinger

**Affiliations:** 1 UMR 1304 GETBO, INSERM, University of Brest, 29200 Brest, France; 2 Internal Medicine, Vascular Medicine and Pneumology Department, Centre Hospitalier Universitaire de Brest, 29200 Brest, France; 3 Department of Medicine I, Clinical Division of Haematology and Haemostaseology, Medical University of Vienna, 1090 Vienna, Austria; 4 Center for Haemophilia Treatment, Haematology, Centre Hospitalier Universitaire de Brest, 29200 Brest, France; 5 Gynecology and Obstetrics Department, Centre Hospitalier Universitaire de Brest, 29200 Brest, France; 6 Gynecology and Obstetrics Department, Morlaix Hospital, 29672 Morlaix, France; 7 CIC1412, INSERM, Centre Hospitalier Universitaire de Brest, 29200 Brest, France; 8 Haemostasis Laboratory, Centre Hospitalier Universitaire de Brest, 29200 Brest, France; 9 CIC-RB Ressources Biologiques (UF 0827), Centre Hospitalier Universitaire de Brest, 29200 Brest, France; 10 Department of Pathology and Laboratory Medicine, UNC Blood Research Center, University of North Carolina at Chapel Hill, NC 27514, USA

**Keywords:** coagulation, plasmin generation, postpartum haemorrhage, predelivery, thrombin generation

## Abstract

**Background**: Postpartum haemorrhage (PPH) is a frequent complication of childbirth that is difficult to predict. Predelivery coagulation biomarkers may help to guide preventive strategies. Our objective was to evaluate the association of predelivery haemostatic biomarkers with non-severe PPH. **Methods:** A nested case-control study was conducted within the « Study of Biological Determinants of Bleeding Postpartum » in order to compare different haemostatic biomarkers in plasma from pregnant women with non-severe PPH (cases) and controls without PPH matched for age, body mass index, term, and mode of delivery. Blood was collected at entry in the delivery room. Global haemostatic assays (thrombin generation assay (TGA) and plasmin generation assay (PGA)) were then performed on freshly thawed aliquots of platelet-poor plasma. **Results:** A total of 370 pregnant women (185 cases and 185 controls) were included. Median [interquartile range] predelivery platelet count was lower in PPH cases than in controls (217 [181–259] versus 242 [196–280] G/L). TGA and PGA parameters were similar between cases and controls. In a subset analysis of vaginal deliveries (n = 144), median predelivery TGA thrombin peak was lower, and median predelivery PGA lag phase was longer in cases compared to controls. In multivariable analysis, only predelivery platelet count was independently associated with non-severe PPH. **Conclusions:** Predelivery platelet count is associated with non-severe PPH. Differences in other haemostatic parameters are tenuous, questioning their usefulness in predicting non-severe PPH.

## 1. Introduction

Postpartum haemorrhage (PPH) is a frequent complication of childbirth that can affect pregnant women worldwide [1,2,3]. The prevalence of PPH is estimated to range from 7 to 25% of deliveries, depending on the definition of PPH, on the mode of evaluation of blood loss, and on the country [4]. Despite the identification of a number of obstetric risk factors for PPH, this complication is still difficult to predict in the general population of delivering women, and targeted preventive strategies are still not uniformly implemented in the maternity wards [5,6]. Most PPH cases are attributed to uterine atony, and the coagulation state of pregnant women prior to delivery has not been extensively investigated as a potential co-factor for bleeding. Most studies have focused on haemostatic biomarkers measured at PPH diagnosis after delivery to guide transfusion procedures or administration of factor concentrates, e.g., fibrinogen [7,8]. In this context, viscoelastic haemostatic assays can be useful to monitor and treat PPH-associated coagulopathy [9,10]. Moreover, the usefulness in preventing PPH would avoid the need for blood product transfusion, emergency surgery, and the negative psychological impact on women experiencing such a complication. That is why we focused our research on the exploration of predelivery haemostatic biomarkers in the context of PPH.

In a previous work within the “Study of Biological Determinants of Bleeding Postpartum” (HPP-IPF), a French cohort study, we found that predelivery endogenous thrombin potential (ETP) measured with the thrombin generation assay (TGA) was lower in women with severe PPH compared to matched pregnant controls, despite similar results in conventional coagulation tests [11].

While severe PPH is defined by a blood loss higher or equal to 1 L (L) at delivery and has a prevalence of around 2% of deliveries, non-severe PPH has a milder clinical phenotype but is much more frequent [4]. As such, the question arises whether predelivery haemostatic biomarkers could therefore help with predicting and possibly preventing this frequent complication of childbirth. 

In this nested case-control study within the HPP-IPF cohort study, we aimed to investigate the association of predelivery haemostatic biomarkers with non-severe PPH. For this purpose, we performed global haemostatic assays (thrombin generation assay (TGA) and plasmin generation assay (PGA)) using plasma samples collected at entry in the delivery room in women who went on to have non-severe PPH and in matched pregnant controls, and evaluated associations of haemostatic biomarkers with non-severe PPH. Since the impact of the haemostatic system might be a more important determinant of bleeding following vaginal delivery versus Caesarean section (C-section), we also evaluated women with vaginal delivery separately in a subset analysis.

## 2. Materials and Methods

### 2.1. Recruitment of Participants, Blood and Data Collection

The HPP-IPF “Study of Biological Determinants of Bleeding Postpartum” is a French prospective single-centre cohort study designed to evaluate the biomarkers associated with PPH among various biological and clinical parameters collected before delivery [12]. All pregnant women entering Brest University Hospital for a delivery between 1 April 2013 and 29 May 2015 were included in the study if they had given their oral consent for the study (Clinicaltrials.gov identifier: NCT02884804). Around 1862 deliveries per year took place at Brest University Hospital during the study period. 

Biological data were measured from a blood sample routinely collected before delivery at entry in the delivery room for blood group determination, complete blood count, and conventional haemostatic tests. The following biomarkers were measured directly in fresh blood samples: haemoglobin, haematocrit, platelet count, immature platelet fraction (IPF) (XE 5000, Sysmex, Villepinte, France), prothrombin rate (STAGO Néoplastine CI + 10, STA-R, Stago, Asnières sur Seine, France), activated partial thromboplastin time (aPTT) ratio (Triniclot Automated APTT, STA-R, Stago, Asnières sur Seine, France), fibrinogen (STAGO STA Fibrinogen 5, STA-R, Stago, Asnières sur Seine, France), D-dimer (STAGO STA Liatest D-Di, STA-R, Stago, Asnières sur Seine, France), and fibrin monomers (STAGO STA Liatest MOFB, STA-R, Stago, Asnières sur Seine, France).

Platelet-poor plasma was prepared after centrifugation (2500× *g*, 15 min), aliquoted, and immediately stored at −80 °C.

Clinical data on women and their pregnancies were collected by midwives and obstetricians in the medical files during each antenatal care visit, from the end of first trimester of pregnancy until delivery, and then in the delivery room, and finally during the maternity stay until discharge. These data were then recorded by trained research assistants in a standardised electronic case report form. Abnormal placental insertion comprised low-lying placentas, placenta previa, placenta percreta, placenta accreta, and placenta bipartita. Antepartum haemorrhage comprised all episodes of uterine haemorrhage occurring at any time during pregnancy, before delivery. The definition of pre-eclampsia was the one provided by the American College of Obstetricians and Gynecologists and published in 2013 [13]. Macrosomia corresponded to a weight of the newborn ≥ 4000 g.

### 2.2. Definition of Non-Severe PPH

Blood loss at delivery was measured systematically with a graduated collector bag. 

Non-severe PPH diagnosis was based on the World Health Organization’s (WHO) definitions of PPH and severe PPH, i.e., a blood loss between 500 and 999 mL in the 24 h following delivery [14].

For each PPH case, the aetiology of PPH was diagnosed by the midwife or the obstetrician in charge of the delivery, according to the “4T” rule [15]. The main aetiology of PPH was recorded if the PPH case was multifactorial. All cases were reviewed and adjudicated by two obstetricians (CaA and CT) in order to confirm the main aetiology of PPH.

### 2.3. Selection of Cases and Controls 

Cases were women included in the HPP-IPF cohort study and diagnosed with non-severe PPH based on the volume of blood loss at delivery. Women for whom the volume of blood loss had not been objectively measured were excluded from the study, as were women delivering before 21 weeks of gestation (WG). Women who had no available frozen plasma sample were also excluded from the study.

Finally, each case was matched with one pregnant woman from the HPP-IPF cohort study as a control with no PPH (i.e., a volume of blood loss < 500 mL at delivery), a delivery at a term ≥ 21WG, and a stored plasma sample. This matching was performed with the support of an experienced data manager (SL) who created a list of potential controls for each case, matched for age at delivery (<35 years versus ≥35 years), pre-pregnancy body mass index (BMI) (<25.0 kg/m^2^ versus [25.0–29.9] kg/m^2^ and ≥30.0 kg/m^2^), term at delivery (<37 WG versus ≥37 WG), and delivery mode (vaginal versus C-section). Then, for each case, CDM selected the pregnant woman without PPH who matched best the PPH case for the four clinical parameters described above. Thus, each non-severe PPH case was matched with the pregnant control woman included in the study who had not presented PPH at delivery and who had the closest clinical characteristics regarding the four matching criteria (age at delivery, pre-pregnancy BMI, term at delivery, and delivery mode). If more than one woman could match one case according to these four matching criteria, CDM chose the control that was the most similar to the case regarding age, BMI, and term at delivery.

The matching of the PPH cases and controls for the main clinical parameters which could represent confounding biases ensures a good reliability of the comparison between non-severe PPH cases and controls, and compensates for the reduced number of controls. This design was also chosen in order to reduce the overall cost of the study and in order to make the measurements feasible by one unique researcher in a period of six months.

### 2.4. Thrombin Generation Assay (TGA)

TGA was performed in plasma using a commercially available kit (Technothrombin, Technoclone, Vienna, Austria) and a low concentration of tissue factor (TF) (PPP Low Reagent, <3 pM final, Stago, Asnières sur Seine, France), as previously described [16]. The following parameters were analysed, using a specifically adapted software (Technothrombin TGA® evaluation software, Biotek KC4, Technoclone, Vienna, Austria, www.technoclone.com): lag phase (minutes), maximum thrombin concentration generated (thrombin peak, nmol/L), time to peak (minutes), velocity index (nmol/L/min), and endogenous thrombin potential (ETP, nmol/L × min).

### 2.5. Plasmin Generation Assay (PGA)

PGA was performed as described [17]. Briefly, two measurements were collected for each plasma sample: one in which endogenous plasmin generation was triggered with a 10 µL solution containing TF, phospholipids, and recombinant tissue plasminogen activator (rtPA), and one in which 10 µL α2M-Pm (calibrator) was added. Plasma was diluted (40 μL of 1:2 dilution in HBS) and added to each well, and the plate was incubated for 10 min at 37 °C. CaCl_2_ and fluorogenic substrate were then dispensed into each well. Final concentrations of reagents were the following: TF (0.5 pmol/L), phospholipids (4 μmol/L), rtPA (0.31 μg/mL), CaCl_2_ (16.6 mmol/L), and fluorogenic substrate (0.5 mmol/L). Measurements were performed with the Calibrated Automated Thrombogram (Stago, Vienna, Austria). Data were analysed as previously described [17]. 

All experiments were performed in a blinded fashion.

### 2.6. Statistical Analysis

To describe the study population, continuous variables are displayed as medians with interquartile ranges (IQR) and categorical variables as counts with percentages. Parameters were compared between non-severe PPH and matched controls without PPH using the Wilcoxon rank sum test for quantitative parameters and Pearson’s chi-squared test for qualitative parameters. They were also compared in a subgroup analysis restricting the cohort to those with vaginal delivery and C-section, respectively. Correlations between quantitative parameters were measured with Spearman’s rank correlation coefficient (ρ). 

The association of haemostatic biomarkers with non-severe PPH was investigated in a logistic regression model, adjusting for clinical parameters described in the literature associated with PPH, and presenting odds ratios (OR) with their 95% confidence intervals (95% CI) [1,2]. We ranked included biomarkers for importance by their “fraction of new information”, that is, the proportion of total predictive information in the full model that was added by the respective biomarker, as described by Harrell [18]. Due to the large number of parameters of interest given the number of events, we conducted principal component analyses of the PGA and TGA parameters, respectively, which were a priori judged to be highly correlated, representing them in the model by their respective first principal component. To further account for the limited number of events per included covariate, we calculated 95% percentile bootstrap confidence intervals running a bootstrap, clustered for matched pairs, for 10,000 iterations. The ability of the final model to discriminate between non-severe PPH and controls was quantified by c-index and Nagelkerke’s R2. Additionally, we visualised the correlation between all biomarkers in a heatmap of their pairwise absolute Spearman’s ρ and using hierarchical clustering of the absolute values of their Spearman’s ρ with a complete agglomeration method.

Missing data were not imputed. All statistical analyses were conducted in R version 4.3.2 using the rms package version 6.7-1 [19,20]. 

### 2.7. Ethics Approval

Pregnant women were informed by midwives and/or obstetricians of Brest University Hospital about the study, and gave their oral consent to participate in the study. The protocol of the study was approved by our institutional review board on 23 August 2011 (Protocol RB 11.080; ID RCB: 2011-A00802-39).

## 3. Results

### 3.1. Characteristics of the Pregnant Women

**Figure 1:**
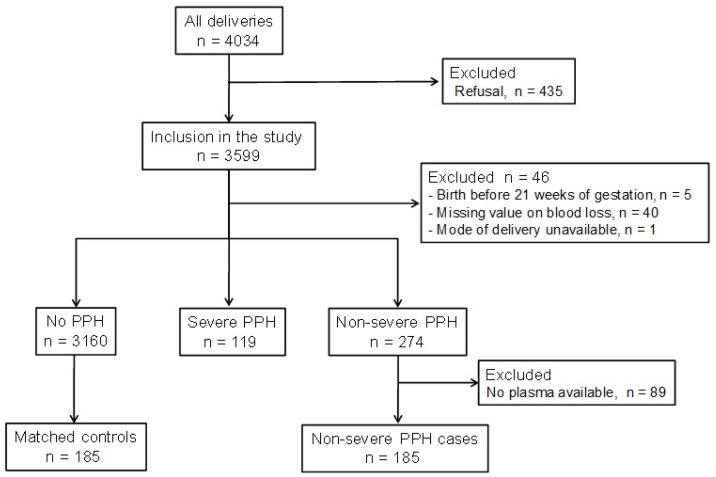
Flow chart of the case-control study. PPH = postpartum haemorrhage.

In this nested case-control study within the HPP-IPF cohort study, 370 pregnant women were investigated: 185 cases who went on to have non-severe PPH and 185 matched pregnant controls (Figure 1). The median [IQR] volume of blood loss in non-severe PPH cases and controls was 600 mL [500–750] and 200 mL [100–300], respectively.

Table 1 illustrates the clinical characteristics of the pregnant women included in this case-control study.

The characteristics of the women and pregnancies between both groups were similar, except for abnormal placental insertion, antepartum haemorrhage, and episiotomy, which were more frequently observed in the non-severe PPH cases compared to controls. 

No pregnant women received anticoagulants the day before delivery, but three pregnant women in the control group were on low-dose aspirin in the days preceding delivery.

Among the 185 non-severe PPH cases, the three main aetiologies of PPH were uterine atony (n = 56, 30.3%), traumatic (n = 54, 29.2%), or placental causes (n = 38, 20.5%). In 39 pregnant women, two PPH aetiologies were combined; and in four, three PPH aetiologies were noted. In 33 (17.8%) PPH cases, no aetiology of PPH could be identified.

### 3.2. Predelivery Haemostatic Biomarkers and Their Association with Non-Severe PPH

Table 2 displays the median values of biomarkers of interest with interquartile ranges, stratified by group.

Pregnant women with non-severe PPH had a lower median [IQR] predelivery platelet count (217 [181–259] versus 242 [196–280] G/L, *p* = 0.003) compared to their matched pregnant controls. Two pregnant women (one case and one control) had a predelivery platelet count below 50 G/L. No significant difference was observed in conventional haemostatic tests. In the subset of women delivering vaginally, we found only weak evidence for lower median [IQR] predelivery platelet count (213 [170–256] versus 234 [192–279] G/L, *p* = 0.06) and median D-dimer levels (1.68 [1.31–2.13] versus 1.56 [1.15–1.86] µg/mL, *p* = 0.05) in non-severe PPH compared to matched controls (Table S1).

### 3.3. Thrombin Generation Assay (TGA)

We found no evidence for a difference in predelivery TGA parameters between non-severe PPH cases and pregnant controls in the full population (n = 370, Table 2). In women delivering vaginally (n = 144, 72 in each group), a decrease in median [IQR] thrombin peak was observed in non-severe PPH cases compared to matched pregnant controls (277.5 [228.6–355.0] versus 317.1 [258.9–410.8] nmol/L, *p* = 0.047) (Figure S1, Table S1).

Over the full population, a weak correlation between blood loss and predelivery TGA time to peak was observed (ρ = 0.11; 95% CI 0.01, 0.21) (Table S2), as it was the case in the subset of women delivering vaginally (ρ = 0.22; 95% CI 0.05, 0.37). In women delivering vaginally, blood loss was also weakly inversely correlated with predelivery TGA thrombin peak (ρ = −0.20; 95% CI −0.36, −0.04) and TGA velocity index (ρ = −0.21; 95% CI −0.36, −0.05).

### 3.4. Plasmin Generation Assay (PGA)

We found no evidence for a difference in predelivery PGA parameters between non-severe PPH cases and matched pregnant controls in the full population. However, in women delivering vaginally (n = 144), median [IQR)] predelivery PGA lag phase was longer in non-severe PPH cases compared to matched controls (2.9 [2.7–3.2] versus 2.7 [2.3–3.0] min, *p* = 0.008) (Figure S2, Table S1).

### 3.5. Logistic Regression Analysis for Parameters Associated with Non-Severe PPH

The linear association of haemostatic biomarkers with non-severe PPH was investigated in a logistic regression model, adjusting for clinical parameters described in the literature associated with PPH.

To reduce the number of biomarkers, we conducted principal component analyses of the PGA and TGA parameters, respectively, representing them in the model with their respective first principal component. 

**Figure 2:**
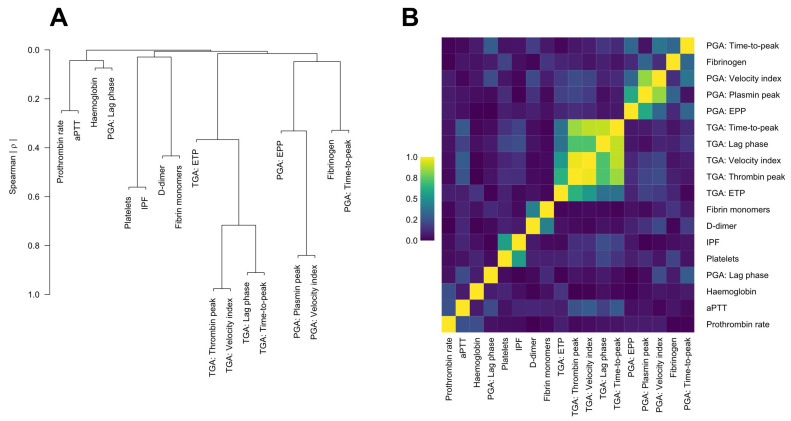
(**A**) Hierarchical cluster analysis of biomarkers of interest using the absolute values of the Spearman correlation coefficients to compute pairwise similarities. (**B**) Heatmap of the absolute values of pairwise Spearman correlation coefficients between biomarkers of interest.

Figure 2 depicts a hierarchical cluster analysis of all biomarkers of interest and a heatmap of their pairwise absolute Spearman’s correlation coefficients. Expectedly, TGA parameters formed a separate cluster, showing high correlation between the curve parameters, albeit less so for ETP. With the exception of the PGA lag phase, which showed barely any correlation with the other curve parameters, PGA presented as a separate cluster with fibrinogen but only modest pairwise correlations, except for PGA plasmin peak and velocity index.

In the logistic regression model presented in Table 3, only two parameters were significantly associated with non-severe PPH: abnormal placental insertion (OR = 3.37; 95% CI 1.06–25.06) and predelivery platelet count. For each decrease in 10 G/L and 50 G/L of predelivery platelet count, the OR of predelivery platelet count for non-severe PPH was 1.06 (95% CI 1.02–1.12) and 1.35 (95% CI 1.10–1.80), respectively. Accordingly, the fraction of new information added to the model was highest for platelets (0.252), which was higher by a factor of 2.7 compared to the second-ranked biomarker, immature platelet fraction (0.094). The concordance index for the model was 0.66 and the Nagelkerke R2 0.11.

## 4. Discussion

In this nested case-control study derived from the French HPP-IPF cohort study, we aimed at evaluating the association of predelivery haemostatic biomarkers with non-severe PPH. Among the parameters that were measured in the study population, only one was significantly different between non-severe PPH cases and matched pregnant controls: the predelivery platelet count. We found no evidence for a difference in predelivery TGA and PGA parameters between non-severe PPH cases and matched controls. However, in pregnant women delivering vaginally, median predelivery TGA thrombin peak was lower and median predelivery PGA lag phase was longer in non-severe PPH cases compared to matched controls. Finally, when incorporating all the haemostatic parameters of interest in a logistic regression model adjusting for established clinical risk factors, predelivery platelet count remained independently associated with non-severe PPH, suggesting a potential role as a predictive biomarker for the assessment of PPH risk at entry in the delivery room.

The platelet count was the sole parameter that remained associated with non-severe PPH among a battery of haemostatic tests in our study. The association of predelivery platelet count with PPH is a consistent and important finding [11,12,21]. The platelet count as part of the blood count analysis is easy to perform and globally widely available. Recently, we published a systematic review and meta-analyses on predelivery haemostatic biomarkers associated with PPH [21]. That review included a total of 81 articles, of which 17 articles were included in the final quantitative synthesis, and various haemostatic parameters. We did not focus on non-severe PPH, but for severe PPH, predelivery platelet count was associated with the clinical outcome [21]. The results of this review are in line with the findings of the present study. 

We did not find any significant difference in predelivery TGA parameters between non-severe PPH cases and controls in the study population. However, in the subgroup of women delivering vaginally, those with non-severe PPH had a slightly but significantly lower thrombin peak compared to matched controls. In a small case-control study focusing on severe PPH, within the same HPP-IPF cohort study, we observed lower predelivery ETP values measured with TGA in pregnant women who developed severe PPH, compared with matched controls [11]. Thus, reduced thrombin generation potential might promote PPH under certain circumstances. We hypothesise that changes in the coagulation profile observed before delivery could be more pronounced in pregnant women who later develop severe PPH than in women who develop non-severe PPH. 

To the best of our knowledge, this is the first study exploring the association of predelivery PGA parameters with non-severe PPH. While we noted no significant difference in predelivery PGA parameters between non-severe PPH cases and matched controls, we detected a difference in predelivery PGA lag phase between cases and controls in the subgroup of pregnant women delivering vaginally. The longer predelivery PGA lag phase observed in non-severe PPH cases delivering vaginally could be interpreted not as a hypofibrinolytic tendency, but as a reflection of the lower predelivery thrombin generation potential, as suggested by their concomitant lower predelivery TGA thrombin peak. 

PPH is a frequent complication of childbirth with many short-term and long-term consequences on women health [22]. Identifying pregnant women at risk for PPH is a key element in preventing this complication, but it remains a challenge. Our study suggests a potential role for predelivery platelet count in predicting PPH. Predelivery platelet count is already used in the USA to assess the risk of PPH at entry in the delivery room [23]. Other countries do not recommend performing a complete blood count prior to delivery. For example, in France, a complete blood count is recommended only at the sixth month of pregnancy, but it is not repeated before delivery in case of physiological pregnancy [24].

However, the role of coagulation in the occurrence of non-severe PPH could also be minor, compared with the role of other factors such as uterine atony. Indeed, uterine atony is the main aetiology of PPH, accounting for approximately 70% of PPH cases [25]. Risk factors for uterine atony are well known, such as obesity, excessive gestational weight gain, multiple pregnancy, macrosomia, labour induction or a prolonged duration of labour [25]. Efforts have been made in the past decade to reduce the occurrence of uterine atony worldwide by the systematic administration of uterotonics after delivery, as recommended by the WHO and the FIGO, but without significant reduction in the incidence of PPH [26,27]. Future research should focus on tools to identify pregnant women at risk for uterine atony and on efficient strategies to manage them appropriately during childbirth in order to avoid the occurrence of PPH. An American case-control study recently published in AJOG by Reitsma et al. performed mass spectrometry on predelivery plasma samples from pregnant women diagnosed with PPH and pregnant controls without PPH matched for age and delivery mode [28]. The authors identified differentially abundant plasma proteins, in particular prostaglandin D2 synthase (PTGDS), which was higher in PPH cases compared with controls [28]. As PTGDS is involved in smooth muscle relaxation and in the inhibition of platelet aggregation, this could be a missing link explaining the association between uterine atony, platelets, and PPH. The authors suggested that the biomarkers they identified as differentially expressed could be incorporated into a score predictive for PPH, which was also our objective in performing this exploratory study.

Our study has some limitations. The monocentric design of the study induced a selection bias, making our results not easily generalizable to other populations of pregnant women. The large number of biomarkers we looked at and the many univariable formal tests and subset analysis we performed led to a highly inflated alpha error and multiple testing issues. However, our study was exploratory and, thus, we refrained from any multiple-testing adjustments; nevertheless, we presented all formal testing that we conducted. The large number of tests thus weakens the evidence for differences found between study cohorts and needs to be acknowledged when interpreting the results. The logistic regression model further includes a large number of biomarkers and clinical covariates. As such, PGA and TGA were represented by their respective first principal component. Moreover, while we did model biomarkers continuously, we did not allow for non-linearity due to sample size limitations, which would be another important research question to investigate. Considering the study design and matching, we further only modelled associations and could not evaluate the predictive performance of the model or biomarkers.

The main strength of our study is the time of blood collection, i.e., at entry in the delivery room, before the clinical bleeding. This prospective design allowed us to investigate the predelivery coagulation profile of pregnant women who later developed non-severe PPH and test for haemostatic biomarkers potentially predictive for non-severe PPH. Second, we collected numerous clinical and biological data on each pregnant woman included in the study, which enabled us to consider a large number of parameters in the risk assessment of non-severe PPH. This study is one of the most comprehensive dealing with haemostatic parameters and the basis for further clinical research in that most important field. 

## 5. Conclusions

In this case-control study of pregnant women from a large prospective cohort, predelivery platelet count was associated with non-severe PPH. Changes in other haemostatic parameters were tenuous in pregnant women later diagnosed with non-severe PPH and may not help for the prediction of PPH. Further prospective observational studies are needed to help with improving the prediction of PPH, what could result in efficient and tailored preventive strategies to avoid PPH events and their consequences on women’s health.

## Figures and Tables

**Table 1:** Clinical characteristics of the 370 pregnant women.

Characteristics	Non-Severe PPH (n = 185)	Controls (n = 185)	*p*
Women’s characteristics			
Age *, years, median (IQR)	30 (26–34)	30 (26–33)	0.54
BMI *, kg/m^2^, median (IQR)	23 (21–27)	23 (21–28)	0.77
25–29.9 kg/m^2^, n (%)	44 (23.8%)	44 (23.8%)	
≥30 kg/m^2^, n (%)	24 (13.0%)	24 (13.0%)	
Smokers, n (%)	51 (27.6%)	58 (31.4%)	0.42
Blood group			
O group, n (%)	82 (44.3%)	86 (46.5%)	0.68
Nulliparous, n (%)	97 (52.4%)	89 (48.1%)	0.41
Pregnancy characteristics			
Conception by ART, n (%)	21 (11.4%)	15 (8.1%)	0.29
Gemellar pregnancy, n (%)	17 (9.2%)	9 (4.9%)	0.10
Gestational diabetes, n (%)	20 (10.8%)	19 (10.3%)	1.0
Gestational weight gain, kg, median (IQR)	13 (8–18)	13 (9–17)	0.85
Pre-eclampsia, n (%)	12 (6.5%)	9 (4.9%)	0.50
IUGR, n (%)	6 (3.2%)	3 (1.6%)	0.51
Placental abruption, n (%)	2 (1.1%)	1 (0.5%)	0.50
Abnormal placental insertion, n (%)	17 (9.2%)	4 (2.2%)	0.003
Antepartum haemorrhage, n (%)	25 (13.5%)	14 (7.6%)	0.01
Delivery characteristics			
Term * at delivery, WG, median (IQR)	39 (37–40)	39 (37–40)	0.77
Induced labour, n (%)	69 (37.3%)	67 (36.2%)	0.83
Vaginal * delivery, n (%)	72 (38.9%)	72 (38.9%)	1.0
Instrumental delivery, n (%)	27 (14.6%)	17 (9.2%)	0.11
C-section *, n (%)	113 (61.1%)	113 (61.1%)	1.0
Emergency C-section, n (%)	77 (41.6%)	91 (49.2%)	0.14
Episiotomy, n (%)	42 (22.7%)	19 (10.3%)	0.001
Perineal tears, n (%)	48 (25.9%)	40 (21.6%)	0.33
Macrosomia, n (%)	17 (9.2%)	16 (8.6%)	0.86

* Matching criteria. ART = Assisted reproductive technology; BMI = body mass index; C-section = Caesarean section; IQR = interquartile range; IUGR = intrauterine growth restriction; PPH = postpartum haemorrhage; WG = weeks of gestation.

**Table 2:** Predelivery haemostatic biomarkers of the 370 pregnant women.

Haemostatic Biomarkers	Non-Severe PPH (n = 185)	Controls (n = 185)	*p*
Blood count parameters			
Haemoglobin, g/dL, median (IQR)	12.1 (11.4–12.8)	12.3 (11.4–12.9)	0.35
Platelets, G/L, median (IQR)	217 (181–259)	242 (196–280)	0.003
IPF, ratio, median (IQR)	5.1 (3.3–7.9)	4.9 (3.1–7.7)	0.63
Conventional haemostatic tests			
Prothrombin rate, %, median (IQR)	100 (94–100)	99 (94–100)	0.73
aPTT, ratio, median (IQR)	1.01 (0.95–1.06)	0.99 (0.94–1.06)	0.12
Fibrinogen, g/L, median (IQR)	4.85 (4.35–5.63)	5.09 (4.46–5.56)	0.32
D-dimer, µg/mL, median (IQR)	1.64 (1.24–2.21)	1.54 (1.08–2.06)	0.06
Fibrin monomers, µg/mL, median (IQR)	5.64 (4.39–9.61)	5.32 (3.98–7.08)	0.10
Thrombin generation assay			
Lag phase, min, median (IQR)	14.1 (12.6–15.1)	14.1 (12.1–15.6)	0.89
Thrombin peak, nmol/L, median (IQR)	294.5 (237.5–389.8)	314.9 (239.3–388.1)	0.57
Time to peak, min, median (IQR)	23.6 (20.6–25.6)	23.1 (20.6–25.6)	0.58
Velocity index, nmol/L/min, median (IQR)	32.6 (22.9–48.3)	35.1 (24.2–49.0)	0.50
ETP, nmol/L × min, median (IQR)	5 419 (4 978–5 919)	5 349 (5 038–5 876)	0.78
Plasmin generation assay			
Lag phase, min, median (IQR)	2.7 (2.6–3.0)	2.7 (2.3–3.0)	0.10
Plasmin peak, nmol/L, median (IQR)	66.8 (52.9–78.0)	67.9 (56.3–78.0)	0.46
Time to peak, min, median (IQR)	7.3 (7.0–8.0)	7.3 (7.0–8.0)	0.67
Velocity index, nmol/L/min, median (IQR)	14.2 (11.9–17.1)	13.8 (11.4–17.0)	0.65
EPP, nmol/L × min, median (IQR)	840.8 (540.6–1 111.8)	844.4 (537.1–1 070.4)	0.87

aPTT = activated partial thromboplastin time; EPP = endogenous plasmin potential; ETP = endogenous thrombin potential; IQR = interquartile range; IPF = immature platelet fraction; PPH = postpartum haemorrhage.

**Table 3:** Logistic regression analysis for biological and clinical parameters associated with non-severe PPH.

Biomarker	25th Percentile	75th Percentile	Difference	OR	95% CI	Fraction of New Information
Platelets (G/L)	187.50	271.50	84.00	0.61	0.37–0.86	0.252
Immature platelet fraction (%)	3.25	7.95	4.70	0.76	0.51–1.05	0.094
aPTT ratio (%)	0.94	1.06	0.12	1.31	0.98–2.02	0.093
Prothrombin time (%)	94.00	100.00	6.00	1.15	0.90–1.51	0.066
D-dimer (µg/mL)	1.16	2.12	0.96	1.10	0.88–1.96	0.024
Haemoglobin (g/dL)	11.40	12.90	1.50	0.92	0.65–1.30	0.009
PC1 plasmin generation assay	−0.92	0.96	1.88	0.93	0.63–1.25	0.007
Fibrinogen (g/L)	4.41	5.58	1.17	0.96	0.65–1.38	0.002
Fibrin monomers (µg/mL)	4.20	8.23	4.04	1.00	0.94–1.05	<0.001
PC1 thrombin generation assay	−0.75	1.12	1.87	0.99	0.72–1.30	<0.001
Adjusted for clinical covariates:						
Age (years)—matched	26.00	33.00	7.00	0.99	0.78–1.24	
BMI (kg/m^2^)—matched	20.70	27.50	6.80	1.08	0.88–1.34	
Nulliparous (yes/no)	No	Yes		1.18	0.73–2.04	
Gemellar pregnancy (yes/no)	No	Yes		2.01	0.62–7.56	
Abnormal placental insertion (yes/no)	No	Yes		3.37	1.06–25.06	
Antepartum haemorrhage (yes/no)	No	Yes		1.51	0.67–3.65	
Pre-eclampsia (yes/no)	No	Yes		0.77	0.14–3.42	
Macrosomia (yes/no)	No	Yes		1.39	0.58–3.58	
Induced labour (yes/no)	No	Yes		1.09	0.67–1.84	
Term at delivery (WG)—matched	37.00	40.00	3.00	1.04	0.80–1.34	
Type of delivery—matched (elective/emergency C-section) (vaginal/emergency C-section)				1.67 1.23	0.78–3.97 0.91–1.66	

BMI = body mass index; CI = confidence interval; C-section = Caesarean section; OR = odds ratio; WG = weeks of gestation.

## Data Availability

The data that support the findings of this study are available from the corresponding author (C.d.M.), upon reasonable request.

## Supplementary Materials

The following supporting information can be downloaded at https://www.mdpi.com/article/10.3390/jcm13144231/s1, Figure S1. Comparison of predelivery TGA thrombin peak in non-severe PPH cases and matched pregnant controls delivering vaginally. The superimposed box plot displays quartiles with whiskers extending to data points 1.5 times the interquartile range; Figure S2. Comparison of predelivery PGA lag phase in non-severe PPH cases and matched pregnant controls delivering vaginally. The superimposed box plot displays quartiles with whiskers extending to data points 1.5 times the interquartile range; Table S1. Predelivery biological characteristics of the 144 pregnant women with a vaginal delivery; Table S2. Correlation between blood loss in mL at delivery and predelivery biomarkers.
