# Supplementary material for: Predelivery Haemostatic Biomarkers in Women with Non-Severe Postpartum Haemorrhage
Source: J Clin Med. 2024 Jul 19;13(14):4231. doi: 10.3390/jcm13144231 (PMC11277716; doi:10.3390/jcm13144231)
Supplement: Supplementary file 1 [file jcm-13-04231-s001.zip › jcm-3045690-supplementary.pdf]

Predelivery Haemostatic Biomarkers in Women with Non-Severe Postpartum Haemorrhage

Claire de Moreuil [1,2,3,*], Brigitte Pan-Petesch [1,4], Dino Mehic [3], Daniel Kraemmer [3], Theresa Schramm [3], Casilda Albert [5], Christophe Trémouilhac [6], Sandy Lucier [7], Hubert Galinat [8], Liana Le Roux [9], Johanna Gebhart [3], Francis Couturaud [1,2], Alisa S. Wolberg [10], Cihan Ay [3] and Ingrid Pabinger [3]

Table S1. Predelivery biological characteristics of the 144 pregnant women with a vaginal delivery

Haemostatic biomarkers	Cases with vaginal delivery (n = 72)	Controls with vaginal delivery (n = 72)	p
**Blood count parameters**			
Haemoglobin, g/dL, median (IQR)	12.3 (11.4-12.9)	12.6 (11.5-13.4)	0.27
Platelets, G/L, median (IQR)	213 (170-256)	234 (192-279)	0.06
IPF, ratio, median (IQR)	5.3 (3.3-8.0)	4.7 (3.2-8.0)	0.73
**Conventional haemostatic tests**			
Prothrombin rate, %, median (IQR)	99 (94-100)	98 (94-100)	0.46
aPTT, ratio, median (IQR)	1.00 (0.94-1.07)	0.99 (0.94-1.05)	0.43
Fibrinogen, g/L, median (IQR)	4.90 (4.50-5.47)	5.07 (4.58-5.63)	0.60
D-dimer, µg/mL, median (IQR)	1.68 (1.31-2.13)	1.56 (1.15-1.86)	0.05
Fibrin monomers, µg/mL, median (IQR)	5.62 (4.41-8.67)	5.32 (3.89-6.68)	0.22
**Thrombin generation assay**			
Lag phase, min, median (IQR)	14.1 (12.6-15.2)	13.6 (12.5-15.1)	0.23
Thrombin peak, nmol/L, median (IQR)	277.5 (228.6-355.0)	317.1 (258.9.-410.8)	0.047
Time to peak, min, median (IQR)	23.6 (21.5-25.1)	22.6 (20.6-25.1)	0.11
Velocity index, nmol/L/min, median (IQR)	30.1 (22.7-43.8)	37.2 (26.1-52.3)	0.06
ETP, nmol/L x min, median (IQR)	5 287 (4 964-5 692)	5 308 (5 014-5 689)	0.84
**Plasmin generation assay**			
Lag phase, min, median (IQR)	2.9 (2.7-3.2)	2.7 (2.3-3.0)	0.008
Plasmin peak, nmol/L, median (IQR)	67.5 (53.5-79.3)	70.6 (59.4-79.4)	0.26
Time to peak, min, median (IQR)	7.3 (7.0-8.0)	7.3 (7.0-7.9)	0.52
Velocity index, nmol/L/min, median (IQR)	14.8 (12.4-17.3)	15.0 (12.2-17.1)	0.75
EPP, nmol/L x min, median (IQR)	839.7 (549.9-1 081.5)	847.7 (603.9-1 058.2)	0.99

aPTT = activated partial thromboplastin time; EPP = endogenous plasmin potential; ETP = endogenous thrombin potential; IQR = interquartile range; IPF = immature platelet function; PPH = postpartum haemorrhage.

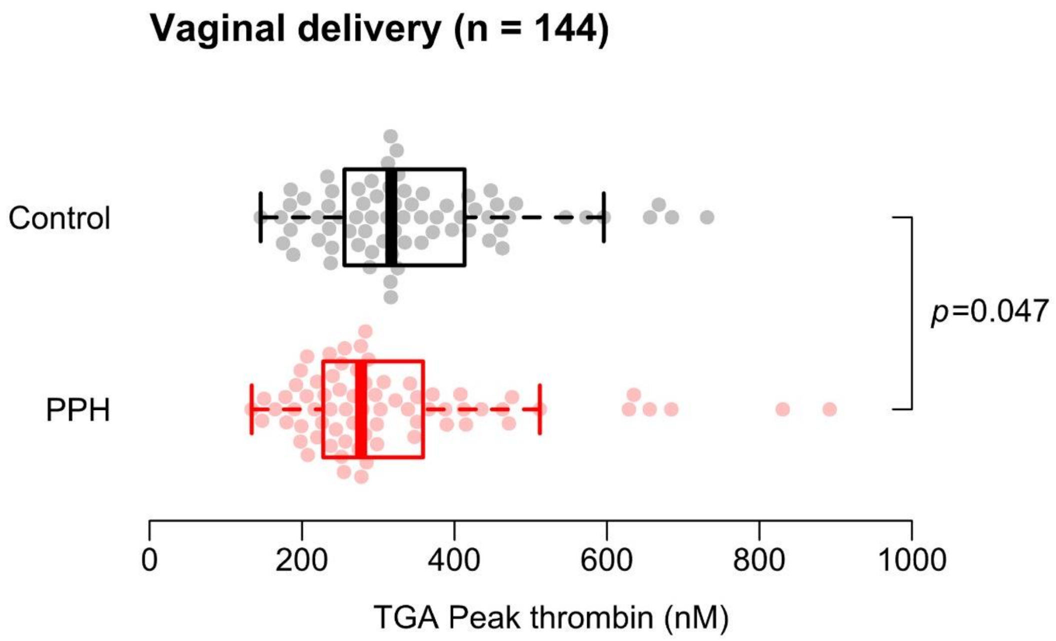

Figure S1. Comparison of predelivery TGA thrombin peak in non-severe PPH cases and matched pregnant controls delivering vaginally. The superimposed boxplot displays quartiles with whiskers extending to datapoints 1.5 times the interquartile range.
PPH = postpartum haemorrhage; TGA = thrombin generation assay

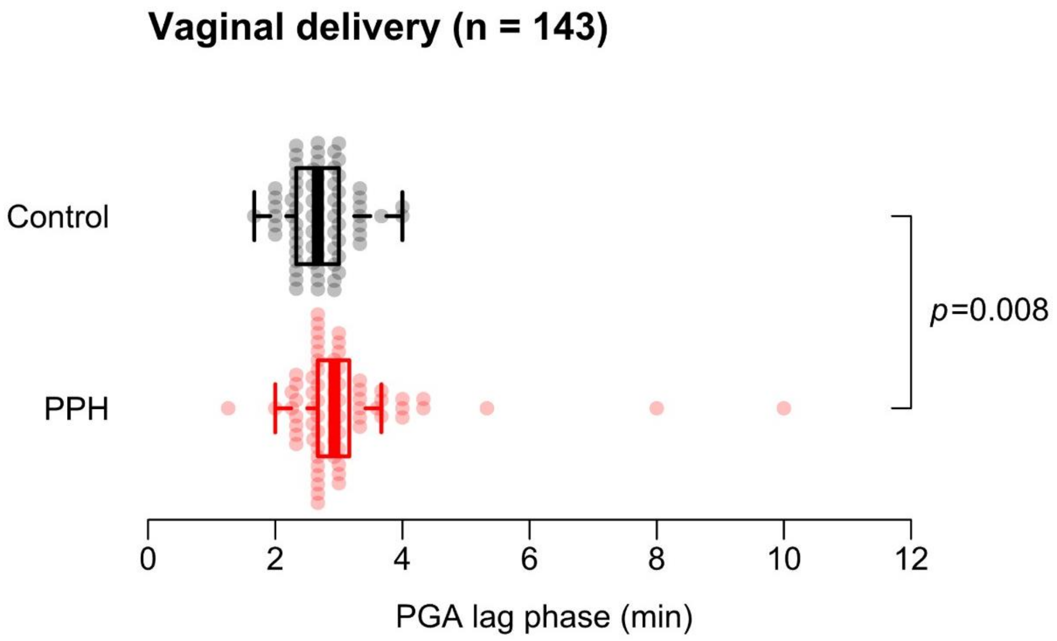

Figure S2. Comparison of predelivery PGA lag phase in non-severe PPH cases and matched pregnant controls delivering vaginally. The superimposed boxplot displays quartiles with whiskers extending to datapoints 1.5 times the interquartile range.
PPH = postpartum haemorrhage; PGA = plasmin generation assay

Table S2. Correlation between blood loss in mL at delivery and predelivery biomarkers

	Full population (n = 370)	
**Haemostatic biomarkers**	**Spearman's ϱ**	**95% CI**
**Blood count parameters**		
Haemoglobin, g/dL, median (IQR)	-0.13	-0.23, -0.03
Platelets, G/L, median (IQR)	-0.12	-0.22, -0.02
IPF, ratio, median (IQR)	0.02	-0.08, 0.12
**Conventional haemostatic tests**		
Prothrombin rate, %, median (IQR)	-0.03	-0.13, 0.07
aPTT, ratio, median (IQR)	0.07	-0.03, 0.18
Fibrinogen, g/L, median (IQR)	-0.07	-0.17, 0.04
D-dimer, µg/mL, median (IQR)	0.13	0.03, 0.24
Fibrin monomers, µg/mL, median (IQR)	0.10	0.00, 0.20
**Thrombin generation assay**		
Lag phase, min, median (IQR)	0.06	-0.04, 0.17
Thrombin peak, nmol/L, median (IQR)	-0.08	-0.18, 0.02
Time to peak, min, median (IQR)	0.11	0.01, 0.21
Velocity index, nmol/L/min, median (IQR)	-0.10	-0.20, 0.01
ETP, nmol/L x min, median (IQR)	0.05	-0.06, 0.15
**Plasmin generation assay**		
Lag phase, min, median (IQR)	0.08	-0.02, 0.18
Plasmin peak, nmol/L, median (IQR)	-0.02	-0.12, 0.09
Time to peak, min, median (IQR)	-0.01	-0.11, 0.09
Velocity index, nmol/L/min, median (IQR)	0.03	-0.07, 0.13
EPP, nmol/L x min, median (IQR)	0.02	-0.08, 0.12
	**Vaginal Deliveries (n = 144)**	
**Haemostatic biomarkers**	**Spearman's ϱ**	**95% CI**
**Blood count parameters**		
Haemoglobin, g/dL, median (IQR)	-0.16	-0.32, 0.01
Platelets, G/L, median (IQR)	-0.14	-0.30, 0.03
IPF, ratio, median (IQR)	0.04	-0.12, 0.21
**Conventional haemostatic tests**		
Prothrombin rate, %, median (IQR)	0.06	-0.11, 0.22
aPTT, ratio, median (IQR)	0.00	-0.17, 0.16
Fibrinogen, g/L, median (IQR)	-0.11	-0.27, 0.05
D-dimer, µg/mL, median (IQR)	0.23	0.06, 0.38
Fibrin monomers, µg/mL, median (IQR)	0.17	0.00, 0.33
**Thrombin generation assay**		
Lag phase, min, median (IQR)	0.16	0.00 0.32

Thrombin peak, nmol/L, median (IQR)	-0.20	-0.36, -0.04
Time to peak, min, median (IQR)	0.22	0.05, 0.37
Velocity index, nmol/L/min, median (IQR)	-0.21	-0.36, -0.05
ETP, nmol/L x min, median (IQR)	0.03	-0.14, 0.19
**Plasmin generation assay**		
Lag phase, min, median (IQR)	0.15	-0.02, 0.31
Plasmin peak, nmol/L, median (IQR)	-0.09	-0.25, 0.08
Time to peak, min, median (IQR)	-0.03	-0.19, 0.14
Velocity index, nmol/L/min, median (IQR)	-0.01	-0.17, 0.16
EPP, nmol/L x min, median (IQR)	-0.05	-0.21, 0.11

aPTT = activated partial thromboplastin time; endogenous plasmin potential = EPP ; endogenous thrombin potential = ETP; interquartile range = IQR ; immature platelet function = IPF ; postpartum haemorrhage = PPH.
